# Highly Sensitized Patients Are Well Served by Receiving a Compatible Organ Offer Based on Acceptable Mismatches

**DOI:** 10.3389/fimmu.2021.687254

**Published:** 2021-06-25

**Authors:** Sebastiaan Heidt, Geert W. Haasnoot, Marissa J. H. van der Linden-van Oevelen, Frans H. J. Claas

**Affiliations:** Eurotransplant Reference Laboratory, Leiden University Medical Center, Leiden, Netherlands

**Keywords:** donor specific antibodies, donor specific antibody (DSA), kidney transplanation, histocompatibility, desensitization, HLA, acceptable antigen, organ allocation

## Abstract

Highly sensitized kidney patients accrue on the transplant waiting list due to their broad immunization against non-self Human Leucocyte Antigens (HLA). Although challenging, the best option for highly sensitized patients is transplantation with a crossmatch negative donor without any additional therapeutic intervention. The Eurotransplant Acceptable Mismatch (AM) program was initiated more than 30 years ago with the intention to increase the chance for highly sensitized patients to be transplanted with such a compatible donor. The AM program allows for enhanced transplantation to this difficult to transplant patient group by allocating deceased donor kidneys on the basis of a match with the recipient’s own HLA antigens in combination with predefined acceptable antigens. Acceptable antigens are those HLA antigens towards which the patients has never formed antibodies, as determined by extensive laboratory testing. By using this extended HLA phenotype for allocation and giving priority whenever a compatible donor organ becomes available, organ offers are made for roughly 80% of patients in this program. Up till now, more than 1700 highly sensitized patients have been transplanted through the AM program. Recent studies have shown that the concept of acceptable mismatches being truly immunologically acceptable holds true for both rejection rates and long-term graft survival. Patients that were transplanted through the AM program had a similar rejection incidence and long-term graft survival rates identical to non-sensitized patients transplanted through regular allocation. However, a subset of patients included in the AM program does not receive an organ offer within a reasonable time frame. As these are often patients with a rare HLA phenotype in comparison to the Eurotransplant donor population, extension of the donor pool for these specific patients through further European collaboration would significantly increase their chances of being transplanted. For those patients that will not benefit from such strategy, desensitization is the ultimate solution.

## Introduction

Sensitization against Human Leucocyte Antigens (HLA) occurs through pregnancy, blood transfusions or organ transplants. Highly sensitized patients awaiting a renal transplant are disadvantaged since their broad immunization status results in positive (virtual) crossmatches with almost all organ donors (1). Such broad immunization status precludes timely transplantation through regular deceased donor allocation schemes, which are based on the exclusion of donors carrying HLA to which the antibodies are directed (unacceptable antigens) (2). In addition, for highly sensitized patients the chance of finding a related or unrelated living donor to which they don’t harbor HLA-specific antibodies is also extremely slim, further reducing their options (3). Highly sensitized patients accrue on the transplant waiting list. Within Eurotransplant, the percentage of patients awaiting a kidney transplant with a Panel Reactive Antibody (PRA) level of ≥85% increased from 2.0% to 5.6% from 2011 to 2019).

One strategy for transplanting highly sensitized patients is to temporarily remove circulating antibodies and/or antibody production by desensitization treatment, creating a window of opportunity for transplantation of either a deceased or living donor organ in the presence of a negative crossmatch (4). While this is a successful procedure for a proportion of highly sensitized patients, it is still hindered by antibody rebound and relatively high acute antibody-mediated, as well as chronic rejection rates (5, 6). In addition, the added burden of immunosuppression involved in such procedures puts the patient at increased risk for infectious complications (pneumonia, BK nephropathy and CMV disease) and malignancies (mainly skin cancer) (6). Finally, these procedures are very costly and resource intensive (7). The survival benefit for patients undergoing desensitization prior to kidney transplantation is not unequivocally clear, since contrasting results have been published (8, 9).

Ideally, one would like to timely transplant highly sensitized patients without administering additional immunosuppressive drugs beyond the standard immunosuppressive protocols. However, this is not possible if the allocation is based on unacceptable antigens. This was realized already in the Netherlands in 1985 when the first Dutch study on developing an alternative program for highly sensitized patients was initiated, which formed the foundation for what we now know as the Eurotransplant Acceptable Mismatch (AM) program (10). Subsequently, the Eurotransplant AM program was officially launched in 1989 with the goal to increase the transplantation rate of highly sensitized patients in the Eurotransplant region (11).

The rationale for the AM program is that by actively defining the acceptable antigens, a negative crossmatch can be predicted. The increased chance for patients to be transplanted in the AM program comes from the addition of the acceptable antigens to the HLA phenotype of the patient, thereby creating an ‘extended’ HLA type on the basis of which allocation takes place, in combination with mandatory shipment of compatible donor organs to the AM patient (12). Patients are transplanted using standard immunosuppressive protocols without additional desensitization treatment. This strategy did not only result in favorable outcomes as discussed below, but is also a cost-effective strategy to transplant highly sensitized patients. Nguyen et al. determined the effect of the AM approach on quality adjusted life years and healthcare costs and showed an overall lifetime gain of 0.034 quality-adjusted life-years and savings of over $4,000 per highly sensitized patient (13). These data imply that the AM approach would be feasible in developing countries as well.

The new Kidney Allocation System (KAS) in the United States, introduced in 2014, was also accompanied with priority for highly sensitized patients within the regular allocation scheme (14). Although 3-year graft survival data of highly sensitized patients transplanted through KAS look promising (15), it remains to be seen if priority without allocation based on acceptable antigens is accompanied by acceptable long-term survival rates. For the Eurotransplant population is has previously been shown that highly sensitized patients that were included in the AM program, but transplanted through regular allocation (exclusion of unacceptable antigens only), had a markedly inferior graft survival compared to highly sensitized patients transplanted through the AM program (16).Therefore, from March this year, patients included in the AM program will only receive organ offers through AM program allocation.

## Defining Acceptable Antigens

Through the years, the AM program has seen many adaptations and updates as technical advances in the field of histocompatibility testing emerged. When the AM program started in the late 1980’s, HLA typing was mainly done by serology, and HLA antibody specificities were determined by using complement dependent cytotoxicity (CDC) assays (17). In those days, acceptable antigens were defined through negative reactions in regular CDC screening assays, but more importantly, by using patient-specific cell panels with single HLA antigen mismatched donor cells for HLA-A and -B, and later, also HLA-DR (18). Again, negative reactions indicated the absence of antibodies against this particular HLA antigen and indicated that a subsequent crossmatch with a donor organ carrying this specific mismatch would be negative. Subsequently, off-the-shelve target cells were generated in the form of Single Antigen Lines (SALs), which are K562 cells transfected with single HLA class I specificities (19). The major advantage of this approach compared to using peripheral blood mononuclear cells or isolated lymphocyte subsets as target cells is that no interference of other HLA alleles is present, but still reactivity to natively expressed HLA antigens is determined (20). More recently, the introduction of solid phase assays, and more specifically the luminex Single Antigen Bead (SAB) assays have facilitated an even more thorough identification of acceptable antigens, especially for HLA class II, for which reliable reagents were historically scarce. Whereas the increased sensitivity, and especially specificity of the SAB assays are useful in this respect, the results of these assays are always interpreted in light of the immunization history of the patient for inclusion in the AM program. Classifying all positive reactions in SAB assays as unacceptable antigens without taking into account specific reaction patterns, the immunization history, the CDC reactions, as well as a thorough risk-benefit analysis for the individual patient, adds to the problem of high sensitization rates rather than solving it (21). Therefore, all applications for the AM program are reviewed by the Eurotransplant Reference Laboratory (ETRL), ensuring equal and transparent inclusion criteria for all patients.

With the field of histocompatibility testing for renal transplantation gradually moving towards HLA epitope matching, the application of epitope analysis for highly sensitized patients was already described before. Computer programs such as HLAMatchmaker allow to extend the repertoire of acceptable antigens through analysis of HLA antigens that do not have epitope mismatches with the total epitope repertoire of the patient’s self HLA (22, 23), and have been used for defining HLA class I acceptable antigens since 2004. Increased knowledge on the relevant epitopes for HLA class II will allow for extending these analyses for HLA class II in the near future (24).

## Transplant Rate of AM Program Patients

**Figure 1:**
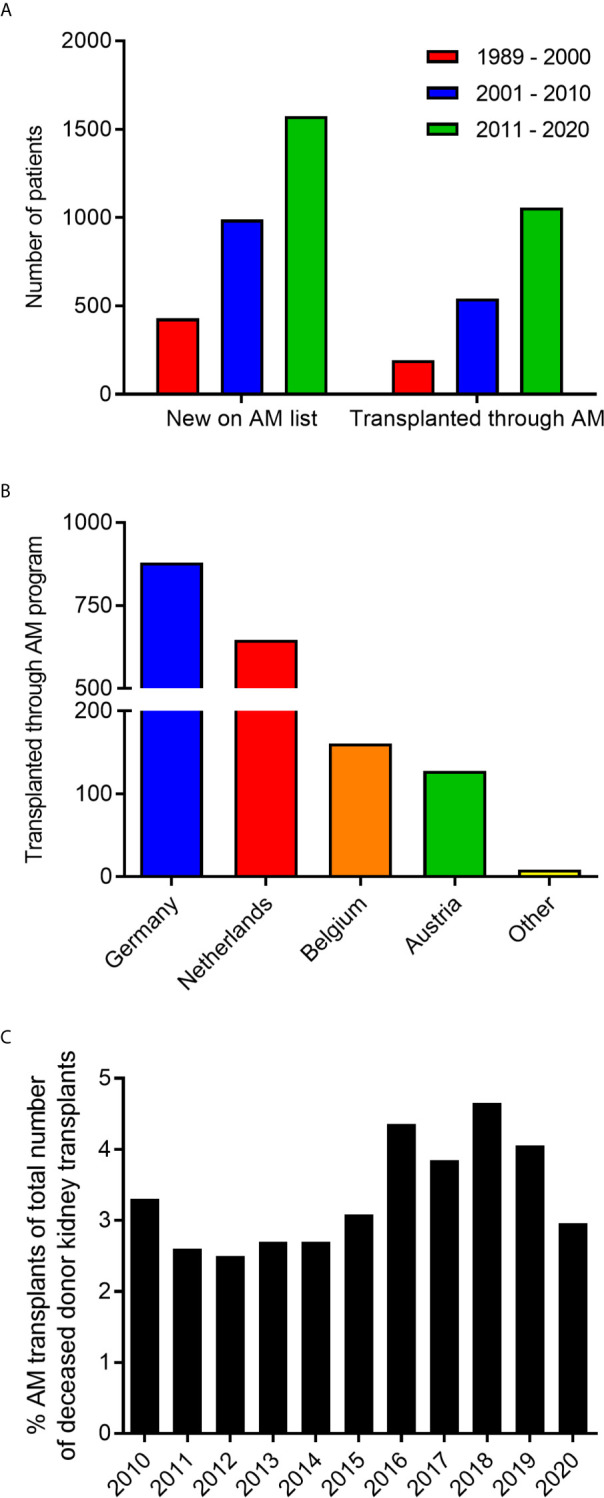
Characteristics of patients enrolled in the AM program. **(A)** The number of patients included in the AM program and transplanted through the AM program from 1989 to 2020. **(B)** Country of origin of patients transplanted through the AM program. **(C)** Percentage of transplants through the AM program within all renal transplants from deceased donors within Eurotransplant in the last 10 years.

Since the start of the AM program in 1989 up till the end of 2020, a total of 2992 highly sensitized patients have been included in the program and 1790 transplants were performed (**Figure 1A**). All countries within Eurotransplant contribute to the AM waiting list and effectuated transplants, with the vast majority coming from Germany and the Netherlands (**Figure 1B**). The numbers for the Netherlands are relatively high because the program started as a Dutch National program and was subsequently extended to the whole of Eurotransplant (12). Of all deceased donor kidney transplants within the Eurotransplant region, on average 3.3% are allocated through the AM program (**Figure 1C**).

**Figure 2:**
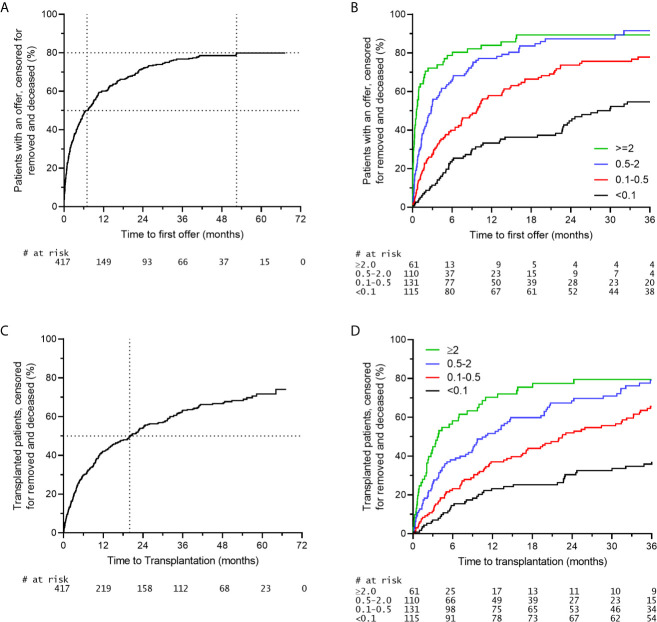
Organ offers and effectuated transplants in the AM program. A time period of 01-01-2015 to 31-21-2016 was selected for inclusion of AM patients (n = 417). **(A)** Rate of first organ offer to patients on the AM waiting list. **(B)** Rate of first organ offer to patients on the AM waiting list stratified for the chance of an organ offer within the AM program. **(C)** Rate of transplantation of patients on the AM waiting list. **(D)** Rate of transplantation of patients on the AM waiting list stratified for the chance of an organ offer within the AM program.

It was previously shown that the waiting time for highly sensitized patients to be transplanted within the AM program is shorter when compared to highly sensitized patients receiving an organ through regular allocation in the Eurotransplant Kidney Allocation System (ETKAS) (16, 22). Since organ offer rates and transplant rates are not necessarily identical, we here analyzed both the organ offer rate and the transplant rate within the AM program. When analyzing the organ offer rate of patients within the AM program it is clear that 50% of patients listed on the AM waiting list receive an offer within the first 7 months of listing. Thereafter, the slope gradually decreases with a plateau of around 80% of patients receiving an offer at 52 months (**Figure 2A**). The rate of organ offers is clearly related to the donor frequency within the AM program, which can be calculated for each AM patient, based on blood group compatibility and the own HLA type plus acceptable antigens (https://etrl.org/FreqAM.aspx) (**Figure 2B**). From these data it is apparent that meticulously defining acceptable antigens contributes to the chance of the individual patients to receive an organ offer within the AM program. Upon analysis of transplants effectuated through the AM program, it is clear that 50% of patients listed receive a transplant within 20 months of listing. After 20 months, AM patients continue to be transplanted with no clear plateau being reached within 67 months (**Figure 2C**). Similar to organ offers, it is clear that patients with the lowest chance as indicated by the donor frequency within the AM program have the slowest rate of transplantation (**Figure 2D**). The disparity between AM offers and effectuated transplants could be due to several reasons, such as patients not being fit for transplant at the time of offer, or the organ not being deemed suitable for the patient involved. It is obvious that immunological reasons, such as institutional minimal match criteria for HLA matching should not result in declining an AM offer, since all mismatches within the AM program are acceptable mismatches, which do not affect graft survival (12, 16).

The data on organ offers and transplantation show that even within priority programs such as the AM program, a subset of patients will not receive any organ offers, especially those in the group of the lowest chance as indicated by the donor frequency within the AM program. These are often patients with a relatively uncommon HLA phenotype in comparison to the available donor population. Here, the rationale that led Professor Jon J. van Rood to initiate the foundation of Eurotransplant, namely the larger the donor population, the higher the chance of finding a compatible donor, still rings true (25). In the proof-of-concept EU-FP7 study entitled ‘A Europe-wide strategy to enhance transplantation of highly sensitized patients on basis of Acceptable HLA mismatches: EUROSTAM’ it was recently shown that up to 27% of highly sensitized patients with neglectable chances of receiving an organ offer within their own donor population had an increased chance of receiving a compatible organ offer from another European allocation system (26). This simulation only included 4 additional donor populations, suggesting an even higher benefit when more organizations would be included. These data advocate the development of an AM program that unites several allocation systems only for those patients that will otherwise not be transplanted.

## Outcome of AM Program Transplants

**Figure 3:**
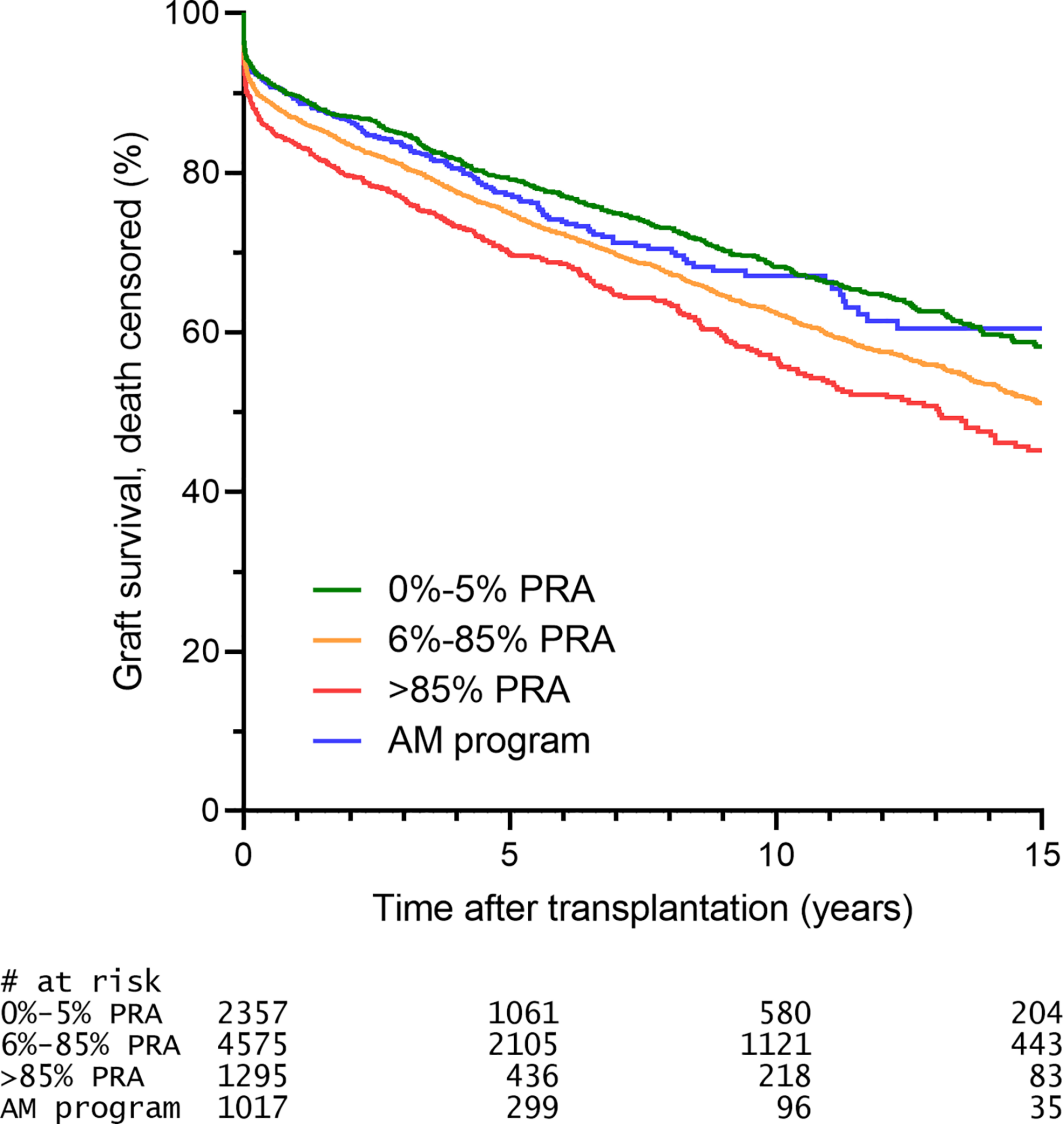
The 15-year death censored graft survival of AM patients is similar to that of unsensitized patients. Selection was based on criteria described prior (16) and included: transplantation from 1996 onwards (start ETKAS allocation), minimum 1 HLA mismatch, kidney only, repeat transplants (since the vast majority of AM patients are repeat transplant candidates). Patients transplanted through ETKAS are subdivided into 0-5% PRA (non-sensitized), 6-85% PRA (intermediately sensitized), >85% PRA (highly sensitized, transplanted outside the AM program).

When the HLA mismatches used for the allocation in the AM program are indeed acceptable, one would expect that there is no effect of these HLA mismatches on graft survival. Indeed, it has been shown that, in contrast to regular allocation, there is no effect of the number of HLA mismatches, either on the broad or split antigen level, on 10-year death-censored graft survival in patients transplanted through the AM program (12, 16). Early studies showed that the 2-year graft survival rates of AM patients were similar to those of non-sensitized patients (22). More recent data with higher patient numbers and longer follow-up showed that patients transplanted through the AM program had similar 10-year death-censored graft survival rates compared to non-sensitized patients transplanted through regular allocation (16). An updated analysis shows that also the 15-year death-censored graft survival is similar when comparing highly sensitized patients transplanted through the AM program and non-sensitized patients transplanted through regular allocation, and significantly better than that of highly sensitized patients transplanted outside the AM program (**Figure 3**). Similar observations regarding short-term graft survival were made in the relatively young Scandiatransplant Acceptable Mismatch Program (STAMP) (27). In their analysis of 96 patients, the authors did observe a non-significant trend towards higher rejection rates in STAMP as compared to control patients. Contrastingly, an analysis of all Dutch patients transplanted through the Eurotransplant AM program (n=113) showed a comparable cumulative rejection incidence to non-sensitized patients, both at 6-months and at 5-years follow-up, whereas highly sensitized patients transplanted through regular allocation had a significantly higher rejection incidence (28). The difference between the outcomes of these two studies could be attributed to the minimal match criterium used in the Eurotransplant AM program and not in the STAMP program, or the fact that the Eurotransplant AM program makes use of a central reference laboratory for strict inclusion into the AM program and acceptable antigen definition, whereas in the Scandinavian program the acceptable antigen definition is performed locally, likely resulting in a less uniform patient population. It has indeed been shown that acceptable mismatch definition by individual centers based on the same serum samples results in a huge variability in what antigens are regarded as being acceptable (29). Other differences between the two programs are that the STAMP program has a minimum waiting time of 1 year before acceptance, whereas the AM program currently has 2 years waiting time as criterium. The minimum PRA for acceptance in STAMP is 80% and currently in the AM program is 85%.

## The Future of the AM Program

As mentioned before, the AM program is continuously adapted to the state-of-the-art knowledge in the field of histocompatibility testing. Some major changes in the AM program are underway that will be discussed here.

### AM Program Inclusion Criteria

The current waiting time before a patient can be submitted to enter the AM program is a generic waiting time (from initiation of dialysis) of 2 years. However, the waiting time for a kidney graft is hugely variable within individual Eurotransplant countries, creating a disbalance between waiting time of highly sensitized patients and all other patients within individual countries, especially those countries with a very long waiting time for regular allocation. Therefore, in the future the median waiting time of a country will be used as minimal time that a patient from that country must be on dialysis before the patient is eligible to enter the AM program. A similar, but separate cut-off will be used per country for pediatric patients. Only for patients with a chance within regular allocation of lower than 0.01% (based on unacceptable antigens fulfilling the criteria below) the waiting time criterium will be omitted.

Traditionally, the AM program was reserved for those patients with a CDC-based PRA of at least 85%. This was later extended to specificities found in luminex, provided that these specificities could be attributed to an immunizing event. With the recent introduction of the vPRA within Eurotransplant the actual chance of receiving an organ offer within regular allocation can be used as inclusion criterium. This chance includes the blood group, as well as the vPRA, based on the HLA phenotype of 10.000 actual organ donors with Eurotransplant. From **Figures 2B, D** it is clear that there is a subgroup of patients that get transplanted within the AM program almost instantly after listing. These patients likely are not that difficult to transplant, warranting stricter inclusion criteria for the AM program. In several publications it has been shown that the patient group that is particularly disadvantaged in regular allocation is the group with a chance of receiving a compatible organ offer of 2% or lower (26, 30). Therefore, the inclusion criterium for the AM program will change to a chance of receiving a compatible organ offer through regular allocation lower than 2%. The unacceptable antigens underlying this value must comply to the ETRL specifications:

-Minimum of one unacceptable antigen must be detectable by CDC.
-The additional unacceptable antigens, defined by Luminex reactivity only, must be attributable to a defined immunizing event.
-In case one of the abovementioned unacceptable antigens results in a clear epitope reactivity pattern, additional antigens carrying this epitope will be included for AM eligibility. The epitopes that will be considered are those that have been indisputably antibody verified, as defined by a list to be published by the ETRL.
-The total list of unacceptable antigens fulfilling the criteria above, together with the blood group must result in an ETKAS chance of <2% for acceptance in the AM program.

### Extension of AM Program Allocation With HLA-C and HLA-DQ

It is clear that allocation to AM patients based on acceptable antigens is superior to allocation to AM patients based on exclusion of unacceptable antigens only (16). However, acceptable antigens currently used for allocation to AM patients are only defined for HLA-A, -B and -DR antigens. With the introduction of solid phase HLA-specific antibody detection techniques it is possible to accurately define acceptable antigens for HLA-C and HLA-DQ as well. In fact, for the vast majority of AM patients, acceptable antigens for HLA-C and -DQ have already been defined but are currently not used for AM allocation. In the near future, allocation will include acceptable HLA-C and -DQ antigens. It is to be expected that outcomes after transplantation will further improve when selection of donors in the AM program is extended to using acceptable antigens for HLA-C and -DQ in addition to HLA-A, -B and -DR.

### Reduction of the Minimal Match Criteria

Currently, within the AM program, minimal match criteria of two HLA-DR or one HLA-DR and one HLA-B (split level) are adhered to. For patients with the chance of an organ offer within the AM program lower than 0.1%, these minimal match criteria are reduced to one HLA-DR match at the broad antigen level. A recent analysis from the ETRL showed that the 10-year graft survival of patients who are transplanted through the AM program according to the minimal match criteria is comparable to those transplanted with reduced minimal match criteria (31). In a second analysis, it was shown that the 6-month cumulative rejection incidence was similar in patients transplanted according to the minimal match criteria and the reduced minimal match criteria (28). These data indicate that acceptable mismatches are truly acceptable and are not detrimental. In a timespan of 2 years, 417 organ offers for AM patients were denied an offer based on the fact that the MMC were not met. Since no effect of the minimal match criteria is found, they will in the future be reduced to one HLA-DR match on the broad antigen level for all AM patients. For patients with the chance of a kidney within the AM program lower than 0.1%, the minimal match criteria will be abandoned altogether.

## Concluding Remarks

Allocation of kidneys to highly sensitized patients remains a challenging task. Over the years, the Eurotransplant AM program has proven to be an efficient way to both prioritize highly sensitized patients, and also maximize transplant longevity. With the fields of histocompatibility and organ allocation evolving, the AM program has been adapted to novel insights and will continue to be updated on basis of the most current insights. Regardless of its success, there is a subset of patients that will not be transplanted through the AM program due to their extremely broad sensitization status in combination with their uncommon HLA phenotypes. For these patients, alternative options must be explored. This could either be looking for compatible donors outside the own donor pool, but could also be by clever integration of living donor programs with priority for highly sensitized patients (32). The latter can include desensitization programs, which represent a valid last resort for those that can otherwise not be transplanted. These include the widely used plasmapheresis and IVIg with or without rituximab, or possibly the more recently introduced complement inhibitor eculizumab, anti-CD20 obintuzumab, or IgG cleaving enzyme imlifidase [reviewed in (33)].

## Author Contributions

SH: wrote the manuscript. GH: performed analyses, co-wrote manuscript. ML-vO: performed analyses, co-wrote manuscript. FC: co-wrote manuscript. All authors contributed to the article and approved the submitted version.

## Conflict of Interest

The authors declare that the research was conducted in the absence of any commercial or financial relationships that could be construed as a potential conflict of interest.

## Abbreviations

AM, acceptable mismatch; CDC, complement dependent cytotoxicity; HLA, Human Leucocyte Antigen; ETKAS, Eurotransplant Kidney Allocation System; ETRL, Eurotransplant Reference Laboratory; KAS, kidney allocation system; PRA, panel reactive antibody; SAB, single antigen bead; AL, single antigen line; STAMP, Scandiatransplant Acceptable Mismatch Program.
